# 基于功能材料的固相微萃取技术及其在法庭科学领域中的应用

**DOI:** 10.3724/SP.J.1123.2022.06018

**Published:** 2023-04-08

**Authors:** Weiya XIE, Xiaohan ZHU, Hongcheng MEI, Hongling GUO, Yajun LI, Yang HUANG, Hao QIN, Jun ZHU, Can HU

**Affiliations:** 1.中国人民公安大学, 北京 100038; 1. People’s Public Security University of China, Beijing 100038, China; 2.公安部物证鉴定中心, 北京 100038; 2. Institute of Forensic Science, Ministry of Public Security, Beijing 100038, China

**Keywords:** 固相微萃取, 法庭科学, 功能材料, 综述, solid phase microextraction (SPME), forensic science, functional materials, review

## Abstract

样品的提取是法庭科学分析中至关重要的一步,尤其是在处理各种复杂基质(如土壤、生物样品、火灾残留物等)中痕量和超痕量的目标分析物时。传统样品前处理技术如索氏提取、液液萃取等,步骤繁琐,耗时耗力,过程中易造成样品的损失和二次污染,且萃取所需的大量溶剂还会对环境及研究人员的健康构成威胁。为此,无需或只需少量溶剂的固相微萃取技术(SPME)应运而生,其小巧便携,操作简单、快速,易于实现自动化等特点使之成为广泛应用的样品前处理技术。早期的研究大多采用商品化SPME装置,然而它的种类有限、价格昂贵、纤维易断、选择性不足,因此,近年来使用金属有机骨架化合物、共价有机骨架化合物、碳基材料、分子印迹聚合物、离子液体、导电聚合物等各种功能材料制备SPME涂层的技术越来越受到关注,在环境监测、食品分析及药物检测等方面应用广泛。然而这些SPME涂层材料在法庭科学领域的应用相对较少,考虑到SPME技术应用于犯罪现场样品原位高效提取的巨大潜力,该文对功能涂层材料进行了简要介绍,系统总结了基于功能材料的SPME技术在炸药、助燃剂、毒品、毒物、油漆、人体气味等分析中的应用,并指出了基于功能材料的SPME技术在法庭科学分析中存在的不足,对其未来可能的发展方向提供了建议,以期为从事相关研究的同行提供参考。

## Abstract (en)

Sample extraction is a crucial step in forensic analysis, especially when dealing with trace and ultra-trace levels of target analytes present in various complex matrices (e. g., soil, biological samples, and fire debris). Conventional sample preparation techniques include Soxhlet extraction and liquid-liquid extraction. However, these techniques are tedious, time-consuming, labor-intensive and require large amounts of solvents, which poses a threat to the environment and health of researchers. Moreover, sample loss and secondary pollution can easily occur during the preparation procedure. Conversely, the solid phase microextraction (SPME) technique either requires a small amount of solvent or no solvent at all. Its small and portable size, simple and fast operation, easy-to-realize automation, and other characteristics thus make it a widely used sample pretreatment technique. More attention was given to the preparation of SPME coatings by using various functional materials, as commercialized SPME devices used in early studies were expensive, fragile, and lacked selectivity. Examples of those functional materials include metal-organic frameworks, covalent organic frameworks, carbon-based materials, molecularly imprinted polymers, ionic liquids, and conducting polymers, all widely used in environmental monitoring, food analysis, and drug detection. However, these SPME coating materials have relatively few applications in forensics. Given the high potential of SPME technology for the in situ and efficient extraction of samples from crime scenes, this study briefly introduces functional coating materials and summarizes the applications of SPME coating materials for the analysis of explosives, ignitable liquids, illicit drugs, poisons, paints, and human odors. Compared to commercial coatings, functional material-based SPME coatings exhibit higher selectivity, sensitivity, and stability. These advantages are mainly achieved through the following approaches: First, the selectivity can be improved by increasing the *π-π*, hydrogen bonds, and hydrophilic/hydrophobic interactions between the materials and analytes. Second, the sensitivity can be improved by using porous materials or by increasing their porosity. Third, thermal, chemical, and mechanical stability can be improved by using robust materials or fixing the chemical bonding between the coating and substrate. In addition, composite materials with multiple advantages are gradually replacing the single materials. In terms of the substrate, the silica support was gradually replaced by the metal support. This study also outlines the existing shortcomings in forensic science analysis of functional material-based SPME techniques. First, the application of functional material-based SPME techniques in forensic science remains limited. On one hand, the analytes are narrow in scope. As far as explosive analysis is concerned, functional material-based SPME coatings are mainly applied to nitrobenzene explosives, while other categories, such as nitroamine and peroxides, are rarely or never involved. Research and development of coatings is insufficient and the application of COFs in forensic science has not yet been reported. Second, functional material-based SPME coatings have not been commercialized as they don’t yet have inter-laboratory validation tests or established official standard analytical methods. Therefore, some suggestions are proposed for the future development of forensic science analyses of functional material-based SPME coatings. First, research and development of functional material-based SPME coatings, especially fiber coatings with broad-spectrum applicability and high sensitivity, or outstanding selectivity for some compounds, is still an important direction for SPME future research. Second, a theoretical calculation of the binding energy between the analyte and coating was introduced to guide the design of functional coatings and improve the screening efficiency of new coatings. Third, we expand its application in forensic science by expanding the number of analytes. Fourth, we focused on the promotion of functional material-based SPME coatings in conventional laboratories and established performance evaluation protocols for the commercialization of functional material-based SPME coatings. This study is expected to serve as a reference for peers engaged in related research.

在法庭科学领域中,调查人员搜集到的样品往往是有限的,且大多存在于复杂基质中,因此样品的采集和制备成了一个至关重要的环节^[1]^。传统样品前处理技术如索氏提取、液液萃取等,步骤繁琐,耗时耗力,过程中易造成样品的损失和二次污染,且萃取所需的大量溶剂还会对环境及研究人员的健康构成威胁^[2⇓-4]^。固相微萃取技术(SPME)无需萃取溶剂、装置小巧便携、操作简单、快速等特点,使之成为法庭科学领域重要的样品前处理技术^[5⇓-7]^。

SPME的萃取原理是基于目标物在基质和涂层之间的分配平衡,因此,涂层类型是影响萃取效率的关键因素^[8]^。目前,商品化的涂层可选择的种类有限,主要包括聚二甲基硅氧烷(PDMS)、聚丙烯酸酯(PA)、二乙烯基苯(DVB)、聚乙二醇(CW),以及上述不同涂层之间的相互组合等^[9]^。商品化的涂层大多是广谱性的,不会对某种或某类特定的分析物具有高选择性。为进一步改善SPME的萃取效率以及对目标化合物的选择性,近年来发展了多种基于功能材料的SPME涂层,如金属有机骨架化合物(MOFs)、分子印迹聚合物(MIPs)、石墨烯(G)、碳纳米管(CNT)、离子液体(ILs)等,在环境监测、食品分析及药物检测等方面应用广泛,检测对象多为挥发性、半挥发性的有机化合物^[8,10]^。然而,在法庭领域科学中,为保障鉴定结论的准确性、客观性,需采用国家标准、行业标准,或经过论证的技术规范来进行分析,对方法的可靠性、重复性要求较高,因此目前采用的仍主要是商品化的SPME涂层,这些基于功能材料的SPME涂层在法庭科学领域的应用相对较少。考虑到SPME技术应用于犯罪现场样品原位高效提取的巨大潜力,本文系统地总结了基于功能材料的SPME技术在法庭科学领域中的应用,以期为从事相关研究的同行提供参考。

## 1 功能涂层材料

为了提高萃取效率以及对目标物的选择性,越来越多的功能材料被用于制备SPME涂层,包括MOFs、共价有机骨架化合物(COFs)、碳基材料、MIPs、ILs、导电聚合物(CPs)等^[11⇓-13]^。

MOFs是由无机金属离子(或金属簇)和有机配体通过配位键自组装形成的多孔杂化材料^[14]^。MOFs具有已知的最高表面积,作为涂层材料具有选择性高、稳定性好、吸附能力强、结构可控、容易调节等优点^[8]^。2009年Cui等^[15]^首次制备了MOF基SPME涂层,用于萃取空气中的苯同系物,萃取效率远高于PDMS和PDMS/DVB涂层。

COFs是一类新兴的由轻元素(B、C、N、O和Si)通过共价键组成的多孔材料^[16]^。COFs具有高比表面积、化学和热稳定性好等优点,且通过对其进行设计和功能化,或与各种材料结合,可以调整其性质,从而对特定目标物达到良好的萃取效果^[17,18]^。Li等^[19]^制备了一种磁性COF(NiFe_2_O_4_@COF)纤维涂层,用于萃取水和人尿液中的抗菌剂三氯生和甲基三氯生,检出限比PDMS/DVB涂层低2~3个数量级。

碳基材料,如G、CNT、富勒烯、有序多孔碳等均已被用作SPME涂层材料^[20]^。碳基材料具有机械性能优、热稳定性强、成本低等特点,通过非共价力与有机分子相互作用,例如氢键、*π-π*堆积、静电力、范德华力和疏水相互作用,可提高对有机化合物(尤其是多环芳烃化合物)的亲和力。2010年Chen等^[21]^首次制备了G基SPME纤维,用于萃取6种拟除虫菊酯类农药,萃取效率是PDMS和PDMS/DVB纤维的1.5倍。

MIPs是在模板分子存在下通过单体与交联剂共聚得到的交联聚合物,包含在大小、形状和化学功能上与模板分子互补的识别位点,对特定化合物具有极佳的选择性^[22]^。2001年首次报道了将MIP应用于SPME的实例,Mullett等^[23]^合成了MIP管内SPME涂层,用于萃取血清中的药品普萘洛尔,即使在其他结构相近化合物的干扰下,该涂层仍能选择性地萃取出普萘洛尔。

ILs是室温下的有机液体盐(熔点低于100 ℃),由有机阳离子和有机/无机阴离子组成^[10]^。ILs通过改变结构,可调整其各种物理和化学性质,从而增强与目标物之间的作用,提高萃取选择性。Aylin等^[24]^制备了一种IL修饰的蒙脱石黏土复合材料,用于萃取水样品中的有机氯农药,检出限比PDMS涂层低2~3个数量级。

CPs是一类将金属电性能和有机聚合物机械性能结合起来的具有高*π*共轭聚合物链的材料^[8]^。常用的CPs有聚苯胺、聚吡咯、聚噻吩及它们之间的复合材料。CPs无需衍生或使用络合剂就可以直接萃取带电物质,扩大了SPME萃取的化合物范围^[8]^。Hajializadeh等^[25]^制备了一种聚苯胺、CNT和沸石咪唑酯骨架的三元纳米复合材料纤维涂层,用于萃取水样中痕量有机污染物,检出限比PDMS涂层低2个数量级。

## 2 基于功能材料的SPME技术在法庭科学领域中的应用

### 2.1 在炸药分析中的应用

爆炸现场基质复杂,干扰严重,且爆炸残留物大多浓度较低,需经过浓缩富集才能检测,SPME技术的发展为解决该问题提供了思路,研究人员制备了许多基于功能材料的高选择性和稳定性涂层用于萃取炸药残留物。

Guan等^[26]^采用涂渍法制备了聚醚砜酮(PPESK)纤维涂层,用于萃取水样中2,4,6-三硝基甲苯(TNT)等7种硝基苯类炸药。PPESK的刚性芳环以及与硝基芳烃的*π-π*、偶极-偶极和极性官能团作用分别赋予该涂层高稳定性和高选择性,检出限比CW/DVB涂层低3个数量级。但涂层的致密表面层阻碍了目标物的转移,导致其内部结构未被充分利用。Harvey等^[27]^采用涂渍法制备了涂有金属*β*-二酮酸盐聚合物的PDMS SPME纤维,用于顶空萃取TNT和2,4-二硝基甲苯(2,4-DNT)。该涂层能够减少基质效应,对TNT和2,4-DNT的萃取富集效果远优于PDMS涂层。Farhadi等^[3]^利用涂渍法制备了碳陶瓷-纳米铜纤维涂层,用于萃取土壤样品中TNT等4种硝基苯类炸药。该涂层的多孔结构赋予其大比表面积,且纳米铜与目标物硝基中的N存在相互作用,提高了选择性和萃取效率。

Bianchi等^[28]^通过胶粘法制备了一种上缘装饰有羧基的喹喔啉腔体(QxCOOHCav)涂层,用于从空气和土壤中萃取TNT等8种硝基苯类炸药。QxCOOHCav与目标物之间存在*π-π*和CH-*π*作用,加之其上缘的羧基与硝基额外的氢键作用,使其能够在大量脂肪烃存在下选择性地萃取硝基苯类炸药。

采用涂渍法和胶粘法制备基于功能材料的SPME涂层虽然简单方便,但由于涂层和基底之间是物理作用,得到涂层的热、化学、机械稳定性较差。为此,Li等^[29]^采用溶胶凝胶技术制备了一种杯芳烃(胺桥杯[4]/羟基硅油)纤维涂层,用于顶空萃取硝酸铵中炸药标记物2,3-二甲基-2,3-二硝基丁烷(DMNB),该涂层与基底之间以化学键连接,因此具有较高的稳定性。另外,杯芳烃中的酰胺键与DMNB之间存在氢键和偶极-偶极作用,与PDMS等商品化涂层相比,萃取能力更强。Mattarozzi等^[5]^制备了一种基于二苯基二乙氧基硅烷(DDP)涂层的平面SPME,用于顶空萃取碎石上的TNT、2,4-DNT和炸药标记物乙二醇二硝酸酯。DDP与目标物的芳环结构和硝基分别存在*π-π*和偶极-偶极作用,比使用商业聚四氟乙烯获得的响应高两倍,且具有优异的热稳定性,加热到400 ℃仍能保持稳定。

除了物理法和溶胶凝胶技术,分子印迹SPME技术等也被用于制备萃取炸药残留物的涂层。Bianchi等^[30]^通过加热聚合法合成了一种以TNT为模板的MIP,以环氧树脂胶粘的方式制备了平均厚度为50 μm的MIP固相微萃取涂层,实现了TNT高灵敏度和选择性的萃取,富集效果相较于商品化的PDMS/DVB涂层高出约两倍。杨瑞琴等^[31]^利用聚酰胺类化合物和气相色谱固定液合成了一种SPME膜,用于萃取土壤水溶液中的TNT。酰胺键和硝基之间存在的氢键作用提高了对TNT的萃取效率,但同时作者也指出受膜吸附容量的限制,该方法只能定性分析,不适于定量分析。Costa等^[6]^将制备的氧化石墨烯(GO)/Fe_3_O_4_磁性纳米复合材料作为分散SPME的吸附剂,该吸附剂将GO出色的萃取能力与Fe_3_O_4_的易于处理相结合,成功用于预浓缩水样中的TNT。

表1总结了基于功能材料的SPME技术在炸药分析中的应用情况。可以看出,目前的研究主要集中于硝基苯类炸药,而应用于过氧化物类炸药的很少甚至没有。这可能是由于硝基苯类炸药的苯环结构更易与功能材料形成*π-π*相互作用而被高效萃取。对于过氧化物类炸药的萃取,目前的研究均是基于商品化的SPME纤维。Muller等^[32]^将PDMS/DVB纤维用于三过氧化三丙酮(TATP)的萃取,采用气相色谱-质谱联用仪(GC-MS)进行检测,检出限为6.4 ng。为进一步提高萃取效率,Wen等^[33]^在活性玻璃纤维过滤器上旋涂溶胶-凝胶PDMS溶液制备了平面SPME萃取装置,将其用于TATP的萃取,采用离子迁移谱进行检测,获得了较高的灵敏度。本研究小组建立了基于PDMS胶粘的SPME涂层制备方法,可制备厚度可控、热稳定性高、机械性能好的石墨烯SPME涂层,用于TATP的萃取,取得了较好的萃取效果。另外,关于涂层方面,MOFs、COFs、ILs等材料在炸药分析中的应用仍需开发。

**表1:** 基于功能材料的SPME技术在炸药分析中的应用情况

Analytes	Sample matrices	Coatings/adsorbents	Coating procedure	Geometries and modes	Detection methods	LODs/ (ng/L)	Ref.
NB, 2-NT, 4-NT, 1,3-DNB, 2,4-DNT, 2,6-DNT, TNT	water	PPESK	dipping	fiber DI-SPME	GC-TSD, GC-ECD	8-126, 0.05-0.59	[26]
TNT, 2,4-DNT	explosives bunker	La(Ⅲ) complex of p-di(4,4,5,5,6, 6,6-heptafluoro-1,3-hexanedionyl) benzene	dipping	fiber HS-SPME	GC-MS	<1 (pg)	[27]
NB, 4-NT, 2,4-DNT, 2,6-DNT, TNT	soil	carbon ceramic copper nanoparticle	dipping	fiber DI-SPME	GC-FID	0.6-2.6 (μg/g)	[3]
NB, 2-NT, 3-NT, 4-NT, 2,6-DNT, 2,4-DNT, 3,4-DNT, TNT	air, soil	QxCOOHCav	physical agglutinating	fiber HS-SPME	GC-MS	4-60, 12.4-38.2 (ng/kg)	[28]
DMNB	ammonium nitrate	amide bridged-C[4]/OH-TSO	sol-gel technology	fiber HS-SPME	GC-ECD	443	[29]
TNT, 2,4-DNT, EGDN	rubble	DDP	sol-gel technology	HS-PSPME	IMS	1.3-4.5 (ng)	[5]
TNT	rubble	MIP with TNT	MISPME	fiber HS-SPME	GC-MS	60	[30]
TNT	soil	polyamides and gas chromatographic stationary solution	-	SPMEM	GC-MS	-	[31]
TNT	water	GO/Fe_3_O_4_	-	DSPME	LC-UV	300	[6]

NB: nitrobenzene; 2-NT: 2-nitrotoluene; 3-NT: 3-nitrotoluene; 4-NT: 4-nitrotoluene; 1,3-DNB: 1,3-dinitrobenzene; 2,4-DNT: 2,4-dinitrotoluene; 2,6-DNT: 2,6-dinitrotoluene; 3,4-DNT: 3,4-dinitrotoluene; TNT: 2,4,6-trinitrotoluene; DMNB: 2,3-dimethyl-2,3-dinitrobutane; EGDN: ethylene glycol dinitrate; PPESK: poly(phthalazine ether sulfone ketone); QxCOOHCav: quinoxaline-bridged cavitand decorated at the upper rim with a carboxyl group; DDP: diethoxydiphenylsilane; MIP: molecularly imprinted polymer; GO: graphene oxide; MISPME: molecularly imprinted solid phase microextraction technology; HS: headspace; DI: direct immersion; PSPME: planar solid phase microextraction; SPMEM: solid phase microextraction membrane; DSPME: dispersed solid phase microextraction; TSD: thermionic specified detector; ECD: electron capture detector; FID: flame ionization detector; IMS: ion mobility spectrometry; UV: ultraviolet detector.

### 2.2 在助燃剂分析中的应用

助燃剂的检测有助于确定火灾事故发生的原因,火灾中常见的助燃剂包括汽油、煤油、柴油等^[34]^。目前,汽油残留物的SPME-GC-MS分析方法已成为法庭科学领域的行业标准(GA/T 1515-2018),本研究小组为该行业标准的主要起草单位,已将该方法用于上百余起案件的检验。尽管该方法已相对成熟,但仍有以下不足,一是商品化SPME涂层萃取效率有限,无法满足陈旧检材中微量助燃剂残留物的检测需求;二是助燃剂残留物的分析难点在于研判,火场基质的干扰严重,助燃剂中特征物质的检验尤为重要,而商品化SPME涂层为广谱性的,因此有必要针对助燃剂中的特征成分建立选择性的萃取方法。

2008年,Ahmad等^[35]^首次将自制的SPME涂层用于萃取纵火分析中的助燃剂。该课题组采用溶胶凝胶技术制备了C_8_涂层用于顶空萃取纵火地毯中汽油、煤油、柴油的所有的烃成分,与PDMS/DVB涂层相比萃取能力和选择性都有所提高。然而,溶胶凝胶技术常用表面易活化的熔融石英为基底,其脆性大,寿命短。为此,Azenha等^[36]^以表面带钛醇基团的刚性钛合金丝为基底,采用溶胶凝胶技术制备了PDMS/SiO_2_混合物纤维涂层,用于顶空萃取火灾碎片上的汽油残留物。刚性的基底和溶胶-凝胶固有特性分别赋予该纤维良好的机械和化学稳定性,且与PDMS纤维相比萃取能力也有所提高。

在之后的研究中,为了进一步提高汽油残留物的萃取效率,Wen等^[37]^以不锈钢丝为基底,采用涂渍法制备了G/ZnO纳米棒(G/ZNRs)纤维涂层,用于从原油中顶空萃取汽油馏分。该涂层有效扩大了G的表面积,并使ZNRs的表面由极性变为非极性,萃取效率是PDMS涂层的2.1~8.2倍。

除萃取汽油顶空的烃成分,许多研究致力于分析汽油中的甲基叔丁基醚(MTBE)。MTBE为汽油中常见的添加剂和抗爆剂,为萃取MTBE, Alizadeh等^[38]^采用电化学沉积法制备了涂有十二烷基硫酸盐掺杂的聚吡咯SPME纤维涂层,用于萃取汽油中的MTBE。该涂层具有多孔结构,热稳定性好,检出限与市售纤维相当,但线性范围有限。之后Amini等^[39]^引入ILs作为涂层材料用于萃取MTBE,采用涂渍法制备了4种基于ILs的SPME纤维涂层,由于ILs与MTBE的疏水相互作用,该涂层显示出良好的萃取效果,且成本很低。然而,该方法制备的涂层与基底之间通过物理作用连接,稳定性较差,为改善其稳定性,该课题组^[40]^利用化学键合法制备了一种与熔融石英基底化学键合的IL纤维涂层,用于顶空萃取MTBE。该涂层和基底之间以化学键连接,具有良好的热稳定性和耐久性。最近,Nims等^[41]^也将ILs涂层材料用于顶空萃取柴油中的芳烃成分,结果显示涂层能够快速、精确且灵敏地提取出苯系物(BTEX),但涂层的可重复利用性差,每提取两次后需要再生。

表2总结了基于功能材料的SPME技术在助燃剂分析中的应用情况。可以看出,对于助燃剂的分析常用顶空SPME模式萃取,因为它们大多具有挥发性。研究主要集中在汽油及其添加剂MTBE,对煤油、柴油等助燃剂的应用较少。关于涂层方面,ILs的应用较多,而MOFs、COFs、MIPs等材料的应用较少甚至没有。

**表2:** 基于功能材料的SPME技术在助燃剂分析中的应用情况

Analytes	Sample matrix	Coatings	Coating procedure	Geometry and mode	Detection method	LOD/ (μg/L)	Ref.
Gasoline, kerosene, diesel	carpet	octyltriethoxysilane/methyltrimethoxysilane	sol-gel technology	fiber HS-SPME	GC-FID	-	[35]
Gasoline	fire debris	PDMS sol-gel mixture/silica particles,	sol-gel technology	fiber HS-SPME	GC-FID	-	[36]
Gasoline	oil	graphene/ZnO nanorods	dipping	fiber HS-SPME	GC-FID	-	[37]
MTBE	gasoline	PPy-DS	electrodeposition	fiber DI-SPME	IMS	-	[38]
MTBE	gasoline	[C_4_C_1_IM][BF_4_], [C_8_C_1_IM][BF_4_], [C_8_C_1_IM][PF_6_], [C_2_C_1_IM][ETSO_4_]	dipping	fiber HS-SPME	GC-FID	0.09	[39]
MTBE	gasoline	1-methyl-3-(3-trimethoxysilyl propyl) imid- azolium bis (trifluoromethylsulfonyl) imide	chemical bonding	fiber HS-SPME	GC-FID	0.1	[40]
BTEX	diesel	[C_8_C_1_IM][PF_6_]	dipping	fiber HS-SPME	GC-MS	-	[41]

MTBE: methyl *tert*-butyl ether; BTEX: benzene, toluene, ethylbenzene, and xylenes; PDMS: polydimethylsiloxane; PPy-DS: dodecylsulfate-doped polypyrrole.

### 2.3 在毒品分析中的应用

法庭科学领域中,检测的毒品大多存在于复杂的生物基质中,如尿液、血浆、头发,为减少基质效应同时提高萃取效率,有必要研发新型SPME涂层。相较于炸药和助燃剂,基于功能材料的SPME技术在毒品分析中的应用更为广泛,许多功能材料制备的SPME纤维涂层已被用于毒品的萃取,包括CPs、ILs、MIPs、MOFs、碳基材料等。

在毒品分析中最常用的CPs是聚吡咯(PPy)。Wu等^[42]^采用化学聚合法制备了PPy管内SPME涂层用于萃取尿液和头发中的苯丙胺(AP)等5种毒品,与使用商业毛细管涂层相比,灵敏度更高。之后,Alizadeh和Bernardo两个研究小组^[43,44]^将PPy作为纤维涂层分别用于萃取血清中甲基苯丙胺(MAP)、3,4-亚甲基二氧甲基苯丙胺和口腔液、尿液中可卡因(COC)等4种毒品,与管内SPME相比,该操作更简单方便,但灵敏度较低。

为提高ILs基涂层的稳定性和可重复利用性,He等^[45]^通过将ILs浸渍的硅氧烷弹性体交联固定在熔融石英纤维表面,制备了可重复使用的ILs基纤维涂层。该涂层利用了硅氧烷弹性体的捕获和支撑作用,具有良好的稳定性和耐久性,并成功用于萃取人尿液中的MAP和AP。

Djozan等^[46]^以二乙酰吗啡(DAMO)为模板制备了一种整体式热稳定的MIP纤维,该纤维克服了传统MIP纤维的脆性和易脱落的缺点,可高效率地萃取水中的DAMO及其结构相似的物质。良好的应用效果使该课题组看到了此方法的应用潜力,因此,他们利用此方法相继开发了基于MAP^[47]^、可待因^[48]^、麻黄碱^[49]^等的MIPs纤维。

作为近年来研究热点的MOFs也被应用于毒品分析中。Shokrollahi等^[50]^通过原位电合成法在阳极氧化的不锈钢丝上制备了基于铜和1,2,4,5-苯四甲酸的MOFs纤维涂层,用于顶空萃取人尿液中的MAP。1,2,4,5-苯四甲酸与MAP存在*π-π*和氢键作用,使涂层具有高选择性。但该涂层制备过程中必须精确控制合成条件,以提高重现性。而Baheri等^[51]^以不锈钢丝为基底采用胶粘法制备了铜基MOFs(HKUST-1)纤维涂层,该方法得到的涂层均匀、稳定且高度可重复,已成功用于萃取生物体液中COC等4种毒品。

碳基材料的种类众多,其中,石墨氮化碳^[52]^、多壁碳纳米管(MWCNTs)^[53]^、GO^[54]^等已成功作为涂层材料用于萃取AP等毒品。Song等^[53]^以不锈钢丝为基底利用胶粘法制备了酸氧化MWCNTs纤维涂层,用于顶空萃取尿液中的苯丙胺类兴奋剂。该涂层具有高表面积,且与苯丙胺类兴奋剂之间存在*π-π*相互作用,萃取效率远高于相同程序制备的PDMS涂层,但文章并未将其与商品化SPME涂层进行比较。

值得注意的是,近年来复合材料越来越受到大家的关注,因为它能结合多种材料的优点从而更好地提取目标物。Hajebi等^[55]^利用静电纺丝技术制备了氧化石墨烯/聚酰胺/聚吡咯纳米纤维(GO/PA/PPy),用于顶空萃取尿液中的MAP。该涂层结合了PA和PPy的稳定性、多功能性和GO的化学、机械稳定性,并且与商业纤维相比更经济。此外,其他研究人员已成功制备并用于萃取毒品的复合材料涂层还包括:碳陶瓷包覆Fe_3_O_4_磁性纳米颗粒^[56]^、MWCNTs/IL^[57,58]^、Fe_3_O_4_/MWCNTs^[59]^等。表3总结了基于功能材料的SPME技术在毒品分析中的应用情况。

**表3:** 基于功能材料的SPME技术在毒品分析中的应用情况

Analytes	Sample matrices	Coatings/adsorbents	Coating procedure	Geometry and mode	Detection method	LODs/ (μg/L)	Ref.
AP, MAP, MDA, MDMA, MDEA	urine, hair	PPy	chemical polymerization method	in-tube SPME	HPLC-MS	8×10^-3^- 56×10^-3^	[42]
MAP, MDMA	serum	PPy-DS	electrodeposition	fiber HS-SPME	IMS	5, 8	[43]
COC, LSD, MAP, MDMA	oral fluid, urine	PPy	electrodeposition	fiber DI-SPME	PESI-MS	0.28-1.33, 1.31-2.65	[44]
MAP, AP	urine	1-ethoxyethyl-3-methylimi- dazloium bis(triflu- oromethane) sulfonylimide	dipping	fiber HS-SPME	GC-MS	0.1, 0.5	[45]
DAMO	water	MIP with DAMO	MISPME	fiber DI-SPME	GC-MS	300	[46]
MAP	saliva	MIP with MAP	MISPME	fiber DI-SPME	GC-FID	14	[47]
Codeine	water	MIP with codeine	MISPME	fiber DI-SPME	GC-MS	-	[48]
Ephedrine, pseudoephedrine	urine, serum	MIP with ephedrine	MISPME	fiber DI-SPME	CE	0.96, 1.1	[49]
MAP	urine	copper-based MOF	in situ electrosyn- thesis	fiber HS-SPME	GC-FID	0.1	[50]
PEP, MAP, AP, COC	water, urine, plasma	HKUST-1	physical aggluti- nating	fiber HS-LSPME	GC-MS	0.1-0.4, 0.2- 0.6, 0.4-0.7	[51]
AP	water	graphitic carbon nitride	dipping	fiber DI-SPME	GC	-	[52]
AP, MAP, MDA, MDMA, MDEA, PTM	urine	MWCNTs	physical aggluti- nating	fiber HS-SPME	GC-MS	0.2-1.3	[53]
AP, MAP	urine	nano graphene oxide sol-gel composite	sol-gel technology	SBSE	HPLC-UV	11, 10	[54]
MAP	urine	GO-PA-PPy	electrospinning	fiber HS-SPME	GC-MS	0.9	[55]
AP, MAP, PEP	urine	ceramic carbon-coated magnetic nanoparticles	sol-gel technology	DI-SBSE	HPLC-UV	5-8	[56]
MAP, ephedrine	water	MWCNTs/IL	dipping	fiber HS-SPME	GC	0.1, 0.07	[57]
MAP, MDMA	urine	MWCNTs/IL	sol-gel technology	fiber HS-SPME	GC-FID	0.097, 0.39	[58]
MAP, ketamine	urine, whole-blood	Fe_3_O_4_/MWCNTs	-	DSPME	GC-MS	0.044, 0.024	[59]

AP: amphetamine; MAP: methylamphetamine; MDA: methylenedioxyamphetamine; MDMA: methylenedioxymethamphetamine; MDEA: 3,4-methylenedioxyethylamphetamine; COC: cocaine; LSD: lysergic acid diethylamide; DAMO: diacetylmorphine; PEP: pseudoephedrine; PTM: phentermine; MOF: metal organic framework; PA: polyamide; MWCNTs: multi-walled carbon nanotubes; IL: ionic liquid; SBSE: stir bar sorptive extraction; PESI: probe electrospray ionization; CE: capillary electrophoresis.

### 2.4 在毒物分析中的应用

在法医毒理学中,从血浆、尿液、胃内容物等复杂基质中提取目标物是几乎所有分析的首要步骤^[60]^。自SPME出现以来,已经被广泛应用于毒理学分析,基于功能材料的SPME涂层应用于农药、苯二氮卓类镇静药物、重金属等毒物萃取的研究亦见报道。

对于有机磷农药,Mehdipour等^[61]^通过计算模拟,设计并合成了MIP聚合物,将其包裹在磁纳米颗粒上,实现了血清中有机磷农药的高效萃取。文章通过对与有机磷农药结构类似的化合物的萃取分析,验证了MIP的选择性。

苯二氮卓类药物在精神疾病的治疗中占有优势地位,然而,这些药物存在于非法市场,导致被许多吸毒者滥用。因此,对人体中此类药物的分析已成为法庭科学的重要任务之一^[62]^。Abrao等^[63]^通过在MIP表面涂渍亲水的牛血清白蛋白层,得到了受限访问分子印迹聚合物(RAMIP)涂层,用于萃取血浆中的苯二氮卓类药物。在适宜pH下,RAMIP表面的蛋白层能与基质中的蛋白质发生静电排斥,避免其进入涂层导致位点的阻塞,从而提高了萃取效率。Kefayati等^[64]^引入了MIP@CuO作为管内SPME的新涂层,与液相色谱联用成功应用于生物样品中苯二氮卓类药物的分析。

在重金属分析中,基于功能材料的SPME技术更多应用于水等环境样品中,而在生物样品中的研究较少。Baile等^[65]^利用ZSM-5沸石和氧化铁磁性纳米粒子的复合材料作为吸附剂,用于萃取尿液样品中的Cd^2+^、Hg^2+^和Pb^2+^。该方法快速、经济、易于处理,但复合材料需要重新改性后才能再次使用。表4总结了基于功能材料的SPME技术在毒物分析中的应用情况。

**表4:** 基于功能材料的SPME技术在毒物分析中的应用情况

Analytes	Sample matrices	Coating/ adsorbent	Coating procedure	Geometry and mode	Detection method	LODs/ (μg/L)	Ref.
Malathion	plasma	ZnFe_2_O_4_-SiO_2_-MIP	MISPME	DSPME	HPLC-PDA	7	[61]
Benzodiazepines	plasma	RAMIP	MISPME	fiber DI-SPME	HPLC-DAD	5-30	[63]
Carbamazepine	water, urine, plasma	MIP@CuO	electrodeposition	in-tube SPME	HPLC-UV	0.01-0.05	[64]
Cd^2+^, Hg^2+^, Pb^2+^	urine	ZSM-5/Fe_2_O_3_	-	DSPME	ICP-OES	0.15-0.20, 0.42-0.73, 0.23-0.39	[65]

RAMIP: restricted access molecularly imprinted polymer; PDA: photo-diode array; DAD: diode array detector; ICP-OES: inductively coupled plasma optical emission spectrometry.

### 2.5 在其他方面的应用

除上述方面,基于功能材料的SPME也被应用于油漆中挥发性成分、人体气味等方面的分析鉴定。

2005年,ILs作为SPME涂层材料被首次应用,Liu等^[66]^采用涂渍法制备了1-辛基-3-甲基咪唑六氟磷酸盐([C_8_C_1_IM][PF_6_])纤维涂层,用于萃取油漆中的BTEX。与市售纤维相比,该涂层成本低,重现性相当,但每次测定后需要重新涂覆,且涂层较薄,灵敏度较低。

Xu等^[67]^以不锈钢丝为基底,采用涂渍法制备了三维绒球状Au/ZnO纤维涂层,用于顶空萃取脚臭中丙酸等6种挥发性脂肪酸(VFAs)。该涂层的多孔结构及其与VFAs的氢键作用,分别赋予其高表面积和高选择性,与二乙烯基苯/碳分子筛/聚二甲基硅氧烷(DVB/CAR/PDMS)涂层相比,表现出更好的萃取效率。同时作者还指出该方法也可应用于腋窝、手、口腔等样本的气味分析。表5总结了基于功能材料的SPME技术在油漆和人体气味中的应用情况。

**表5:** 基于功能材料的SPME技术在油漆和人体气味中的应用情况

Analytes	Sample matrices	Coating	Coating procedure	Geometry and mode	Detection method	LODs	Ref.
BTEX	paints	[C_8_C_1_IM][PF_6_]	dipping	fiber HS-SPME	GC-FID	0.1-0.8 μg/mL	[66]
Propanoic acid, butyric acid, isobutanoic acid, isovaleric acid, hexanoic acid, heptylic acid	foot odor	three-dimensional pompon-like Au/ZnO porous microspheres	dipping	fiber HS-SPME	GC-MS	0.017-0.098 ng	[67]

## 3 结论

近年来,已研发了多种基于功能材料的SPME涂层并被广泛应用,包括MOFs、COFs、ILs、CPs、MIPs等。相较于商品化涂层,这些涂层的选择性和灵敏度更高、稳定性更好。而其优势主要通过以下途径实现:(1)增加涂层和目标物之间的*π-π*堆积、氢键、亲水/疏水等相互作用提高选择性。(2)使用具有多孔结构的材料或增大材料的孔隙率以增加比表面积提高灵敏度。(3)提高涂层自身刚性或使涂层与基底之间以化学键连接提高热、化学和机械稳定性。此外,兼备多重优点的复合材料正逐步替代单一材料应用于SPME涂层的制备。在基底方面,金属支撑正逐步替代熔融石英支撑。

然而,基于功能材料的SPME技术在法庭科学领域的应用仍较为有限。一是分析对象有限,如在炸药分析方面,基于功能材料的SPME涂层主要应用于硝基苯类炸药,而涉及其他类别如硝胺类、过氧化物类的很少甚至没有。二是涂层的研发利用不够,COFs等涂层材料在法庭科学领域的应用尚未见报道。此外,基于功能材料的SPME涂层还没有经过实验室间的验证或建立官方标准分析方法,导致未实现商业化。因此,要将基于功能材料的SPME技术应用于法庭科学分析,未来可能的发展方向有:(1)紧密结合材料领域的最新成果,继续研发新型涂层,尤其是具有广谱性和高灵敏度,或对某些化合物具有突出选择性的涂层。(2)引入分析物与涂层之间结合能的理论计算,以指导功能涂层的设计,提高新型涂层筛选的效率。(3)拓展分析对象,扩大在法庭科学领域中的应用。(4)注重新型涂层在常规实验室的推广,建立性能评价方案,为基于功能材料的SPME涂层的商业化铺平道路。
